# Induction of systemic resistance through calcium signaling in Arabidopsis exposed to air plasma-generated dinitrogen pentoxide

**DOI:** 10.1371/journal.pone.0318757

**Published:** 2025-02-06

**Authors:** Shota Sasaki, Hiroto Iwamoto, Keisuke Takashima, Masatsugu Toyota, Atsushi Higashitani, Toshiro Kaneko

**Affiliations:** 1 Graduate School of Engineering, Tohoku University, Sendai, Japan; 2 Department of Biochemistry and Molecular Biology, Saitama University, Saitama, Japan; 3 Suntory Rising Stars Encouragement Program in Life Sciences (SunRiSE), Suntory Foundation for Life Sciences, Soraku-gun, Kyoto, Japan; 4 College of Plant Science and Technology, Huazhong Agricultural University Wuhan, Hubei, China; 5 Graduate School of Life Sciences, Tohoku University, Sendai, Japan; 6 Division for the Establishment of Frontier Sciences of the Organization for Advanced Studies, Tohoku University, Sendai, Japan; Hainan University, CHINA

## Abstract

Plasma technology, which can instantaneously transform air molecules into reactive species stimulating plants, potentially contributes to developing a sustainable agricultural system with high productivity and low environmental impact. In fact, plant immunity activation by exposure to a reactive gas mainly consisting of dinitrogen pentoxide (N_2_O_5_) was recently discovered, while physiological responses to N_2_O_5_ are rarely known. Here, we demonstrate early (within 10 min) physiological responses to N_2_O_5_ gas in Arabidopsis. Exposure to N_2_O_5_ gas induced an increase in cytosolic Ca^2+^ concentration within seconds in directly exposed leaves, followed by systemic long-distance Ca^2+^-based signaling within tens of seconds. In addition, jasmonic acid (JA)-related gene expression was induced within 10 minutes, and a significant upregulation of the defense-related gene *PDF1*.*2* was observed after 1 day of exposure to N_2_O_5_ gas. These systemic resistant responses to N_2_O_5_ were found unique among air-plasma-generated species such as ozone (O_3_) and nitric oxide (NO)/nitrogen dioxide (NO_2_). Our results provide new insights into understanding of plant physiological responses to air-derived reactive species, in addition to facilitating the development of plasma applications in agriculture.

## Introduction

Atmospheric pressure plasma (APP) technology, which allows for a strong non-equilibrium chemical reaction initiated by high-energy electrons, is capable of efficient chemical conversion [1–5]. Specifically, air APP can convert nitrogen (N_2_), oxygen (O_2_), and water (H_2_O) molecules on-site into gaseous reactive species H_x_N_y_O_z_ [for example, ozone (O_3_), nitric oxide (NO), nitrogen dioxide (NO_2_), and hydroxyl radical (OH)], which are useful for medical [6–15], agricultural [16–21], and environmental applications [22, 23], potentially increasing the utilization of air to a great extent. Deemed a promising next-generation solution in these fields, this technology, which is capable of operating exclusively with ambient air and distributed renewable energy, such as solar power, may stand out for its environmental sustainability and versatility. In recent years, potential applications such as sterilization/virus inactivation [24–32], plant growth promotion [33–35], plant immune activation [36], nitrogen fertilization [16, 17, 20], seed germination promotion [34, 37, 38], and food preservation [39, 40] have been extensively investigated, and it has been reported that reactive oxygen species (ROS) and reactive nitrogen species (RNS) can play an important role in these processes. On the other hand, precise control of the H_x_N_y_O_z_ gas composition is still challenging, and many studies have used mixtures of several H_x_N_y_O_z_ (*e*.*g*., a mixture of O, O_3_, and HNO_3_). Consequently, ROS and RNS stress to plants have hardly been controlled and have not been quantified or compared; therefore, the development of the air APP applications faces the challenge of unveiled mechanism of action of APP-synthesized H_x_N_y_O_z_ on biological targets (plants, seeds, microorganisms, etc.).

Plants are constantly subjected to a wide range of biotic and abiotic stresses from the environment [41–44]. To adapt to these stresses, plants exhibit a multitude of responses to such stresses, from gene expression to physiology and from changes in plant architecture to primary and secondary metabolism. Well-known plant responses include responses to mechanical stresses such as wounding, which change gene expression patterns and are mediated by hormones such as jasmonic acid (JA), ethylene (ET), salicylic acid (SA), and abscisic acid (ABA) [45]. More recently, our co-author discovered that systemic propagation of Ca^2+^ signaling from a wounded leaf is triggered within minutes by glutamate-based systems in Arabidopsis [46]. This leads to the activation of JA-induced defense responses. On the other hand, plants also respond to chemical stresses including gaseous exogenous reactive species. The plant response to gaseous O_3_ [47–50], for which the selective synthesis method using APP was first reported in 1857 [3], has been intensively investigated in gaseous H_x_N_y_O_z_. Many reports generated O_3_ based on APP technology and showed that O_3_ exposure at relatively low-density ranges (< 1 ppm) and in the long term (from hours to days) elicited various responses, including a rapid increase in cytosolic Ca^2+^ concentration ([Ca^2+^]_cyt_) as the earliest response and activation of salicylic acid-dependent signaling pathways [47, 51]. However, to the best of our knowledge, there are few reports on plant responses to other H_x_N_y_O_z_ combinations, except for some reports on exogenous NO and NO_2_ [52–56].

We engaged in composition control and quantification of H_x_N_y_O_z_ generated in air APP-activated gas [27, 28, 57] and reported a new APP system for the selective synthesis of dinitrogen pentoxide (N_2_O_5_) a few years ago [58], in addition to existing plasma synthesis of O_3_ and NO/NO_2_ from air. Furthermore, the activation effects of APP-synthesized N_2_O_5_ gas on plant immunity were demonstrated through pathogen inoculation tests on N_2_O_5_-stimulated plants, indicating its potential as a new technology for controlling plant diseases [59]. However, the biological mechanisms by which plants respond to dinitrogen pentoxide at the fundamental physiological level remain poorly understood. In the present study, we investigated real-time responses in plants exposed to selectively synthesized N_2_O_5_ compared to O_3_ and NO/NO_2_, focusing on Ca^2+^ signaling and the expression of defense-related genes. This study is the first report on the N_2_O_5_-induced local and systemic Ca^2+^ responses in plants, providing valuable insights into plant physiology in response to exogenous gaseous ROS and RNS, potentially useful for the development of sustainable agriculture using air APP.

## Results

### Construction of a plant N_2_O_5_ exposure system with live imaging of [Ca^2+^]_cyt_

**Fig 1:**
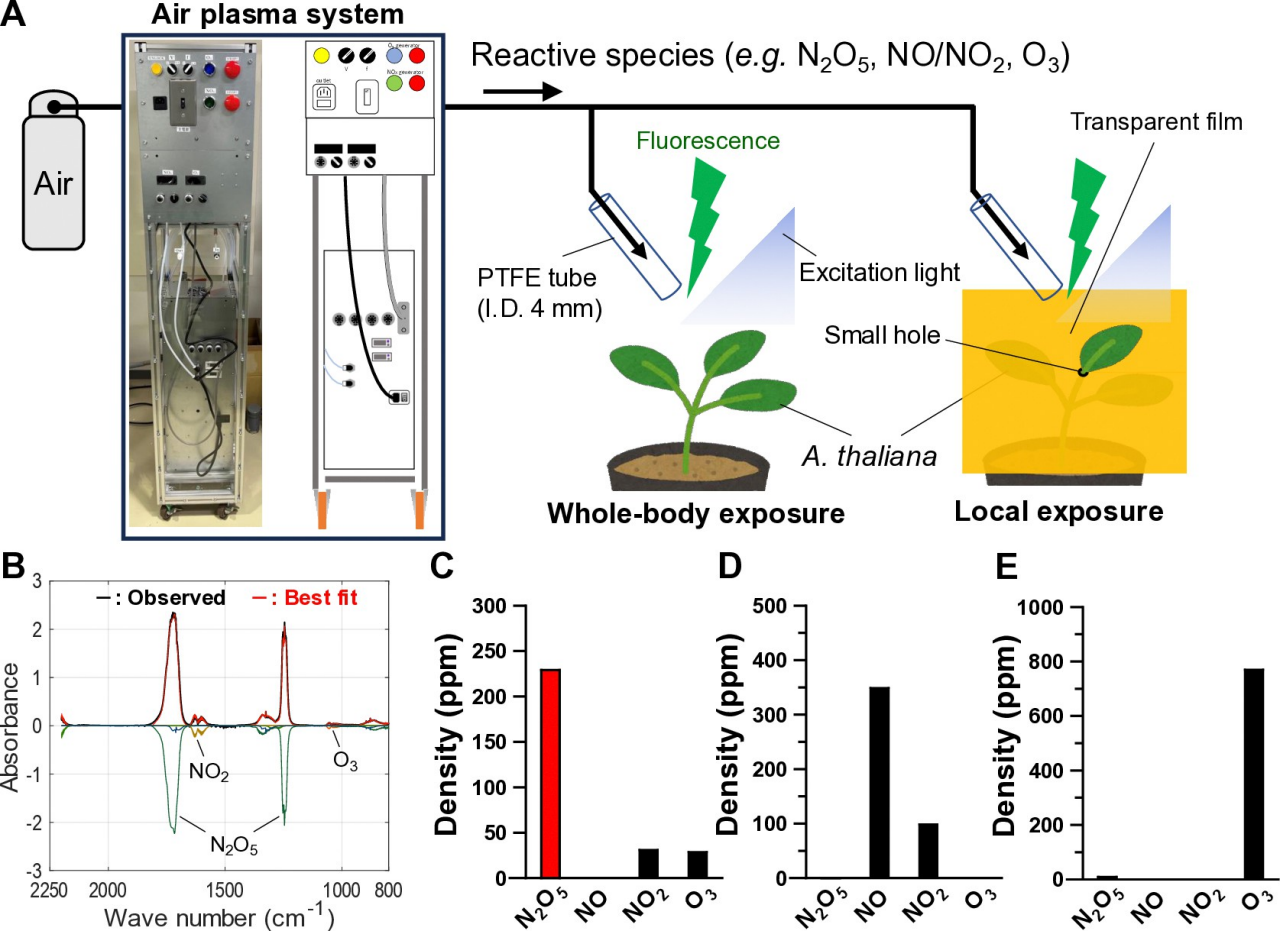
On-site generation of high-density reactive species using air plasma technology and live-imaging of [Ca^2+^]_cyt_ in *A*. *thaliana* plants stimulated by reactive gases. (A) Experimental schematic illustration of [Ca^2+^]_cyt_ imaging in plants after whole-body and local exposure to air plasma-generated reactive species. (B) Typical FT-IR absorbance spectrum of a reactive gas in the N_2_O_5_ mode. The absorbance spectra of individual species composing the best-fit synthetic spectrum for the observed spectrum are shown as negative absorbance spectra. Reactive species’ composition of reactive gases in (C) N_2_O_5_ mode, (D) NO_x_ mode, and (E) O_3_ mode.

N_2_O_5_ gas, which cannot be stored in ambient air, was synthesized using a newly built transportable device consisting of the plasma module (Fig 1A), two mass flow controllers, electric control components, and a mixing reactor, using the same mechanism as in the previous study [58]. The electric switches on the device determine reaction processes in the flow reactor in a mode to provide a desired gas composition consisting of N_2_O_5_, NO_x_ (defined as a mixture of NO and NO_2_), and O_3_. The reactive gas was sprayed onto the whole-body or a single leaf of *Arabidopsis thaliana* plant. The local treatment of a leaf was conducted by covering the plant with a transparent film except for the targeted leaf.

Fig 1B–1E show the gas compositions continuously generated from air are controlled at the device exit. In a reaction process mode for N_2_O_5_ generation (Fig 1C), the densities of N_2_O_5_, NO_2_, and O_3_ were approximately 230, 29, and 31 ppm, corresponding to 0.32, 0.041, and 0.044 μmol/s, respectively. In the NO_x_ mode (Fig 1D), the densities of NO and NO_2_ were approximately 350 and 99 ppm, respectively, corresponding to 0.49 and 0.052 μmol/s, respectively. In O_3_ mode (Fig 1E), the O_3_ density was approximately 770 ppm, corresponding to 1.1 μmol/s. Details are provided in S1 Table. Note that the APP-synthesized N_2_O_5_ gas contained low levels of NO_2_ and O_3_ as impurities due to the unavoidable generation and decomposition reactions involved in N_2_O_5_ chemistry, and the effects of N_2_O_5_ gas should be carefully interpreted. The reactive gases mixtures in the N_2_O_5_, NO_x_, and O_3_ modes were hereafter labeled as “N_2_O_5_ gas,” “NO_x_ gas,” and “O_3_ gas,” respectively. Live imaging of [Ca^2+^]_cyt_ was conducted with transgenic *A*. *thaliana* expressing the GFP-based Ca^2+^ indicator, GCaMP3, and a fluorescence stereo microscope [44, 46, 60].

### Plant damage due to exposure to reactive gases in N_2_O_5_, NO_x_, and O_3_ modes

**Fig 2:**
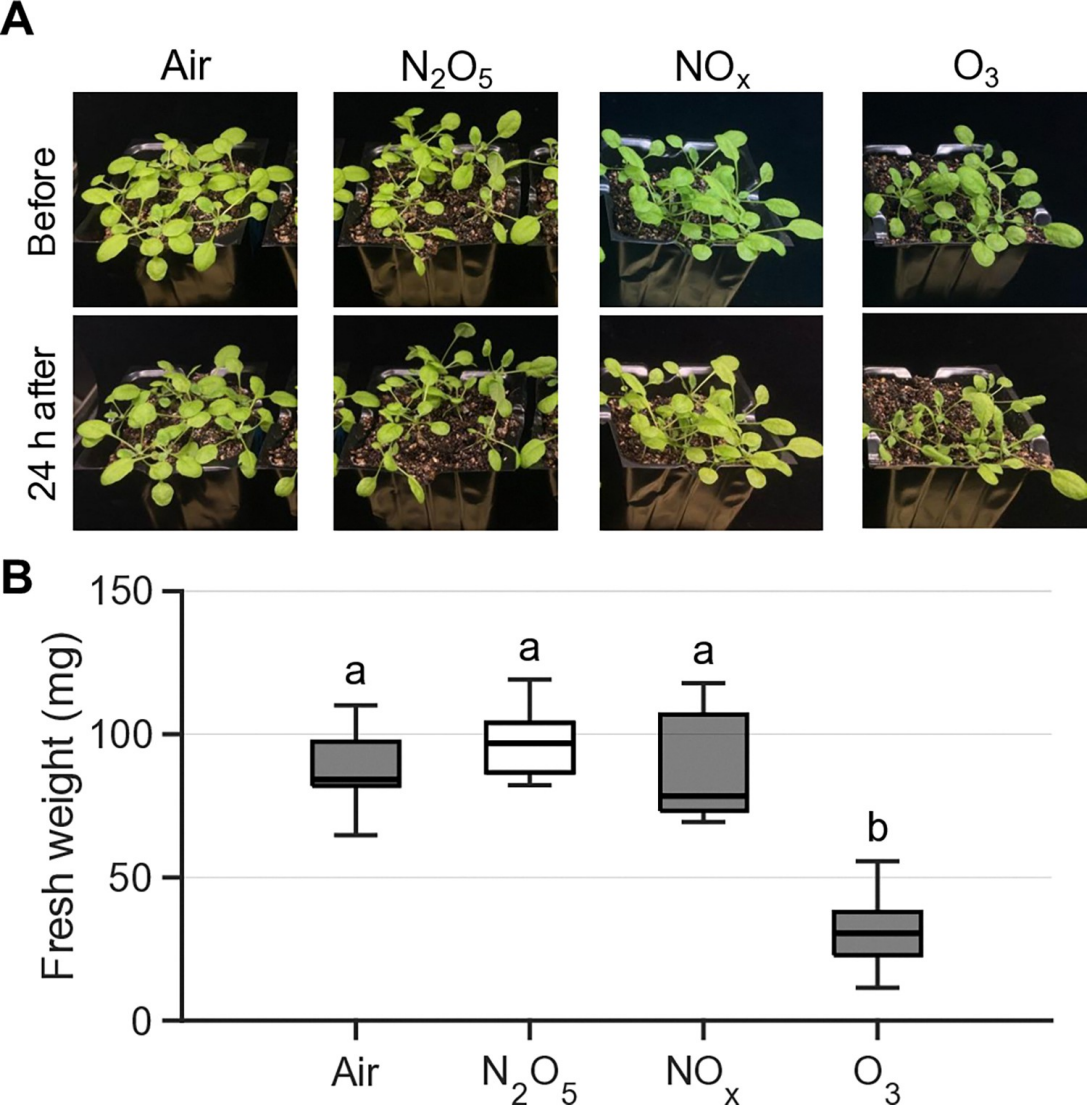
Observation of plant damage after exposure of plants to reactive gases. (A) Typical images of plants (two-week-old Col-0) at 24 hours and (B) fresh weights at 2 weeks after exposure to dry air, N_2_O_5_, NO_x_, and O_3_ gases. Statistical analysis was performed with Tukey-Kramer test (n = 7, p < 0.01). The treatment time was 30 s for dry air and N_2_O_5_, 15 s for NO_x_, 10 s for O_3_, corresponding to a total dose of approximately 10 μmol for N_2_O_5_, NO+NO_2_, and O_3_.

Short-duration (10 s) exposure to O_3_ gas caused immediate and obvious acute injury to the plant within the first few seconds: leaves were hanging pendulously and edges were curling, giving leaves a wilted appearance, well known acute toxicity symptoms of O_3_ [47, 61]. At 24 hours (Fig 2A) and 2 weeks (S1 Fig) after the O_3_ exposure treatment, plant damage was clearly observed, and the fresh weight was significantly lower after 2 weeks (Fig 2B). In contrast, N_2_O_5_ and NO_x_ gas exposure, despite the similar total dose (~ 10 μmol) to O_3_ gas exposure, showed no apparent damage after 24 hours (Fig 2A), and the fresh weight after 2 weeks was almost the same as that of the control sample exposed to dry air (Fig 2B). This clearly shows that the O_3_-induced toxicity to plants is very high compared to that of N_2_O_5_ and NO_x_. Thus, N_2_O_5_ was found less phytotoxic reactive species than O_3_, at least under such high-density and short-duration exposure conditions.

### Ca^2+^ signaling induced by the APP-synthesized reactive gases in N_2_O_5_, NO_x_, and O_3_ modes

**Fig 3:**
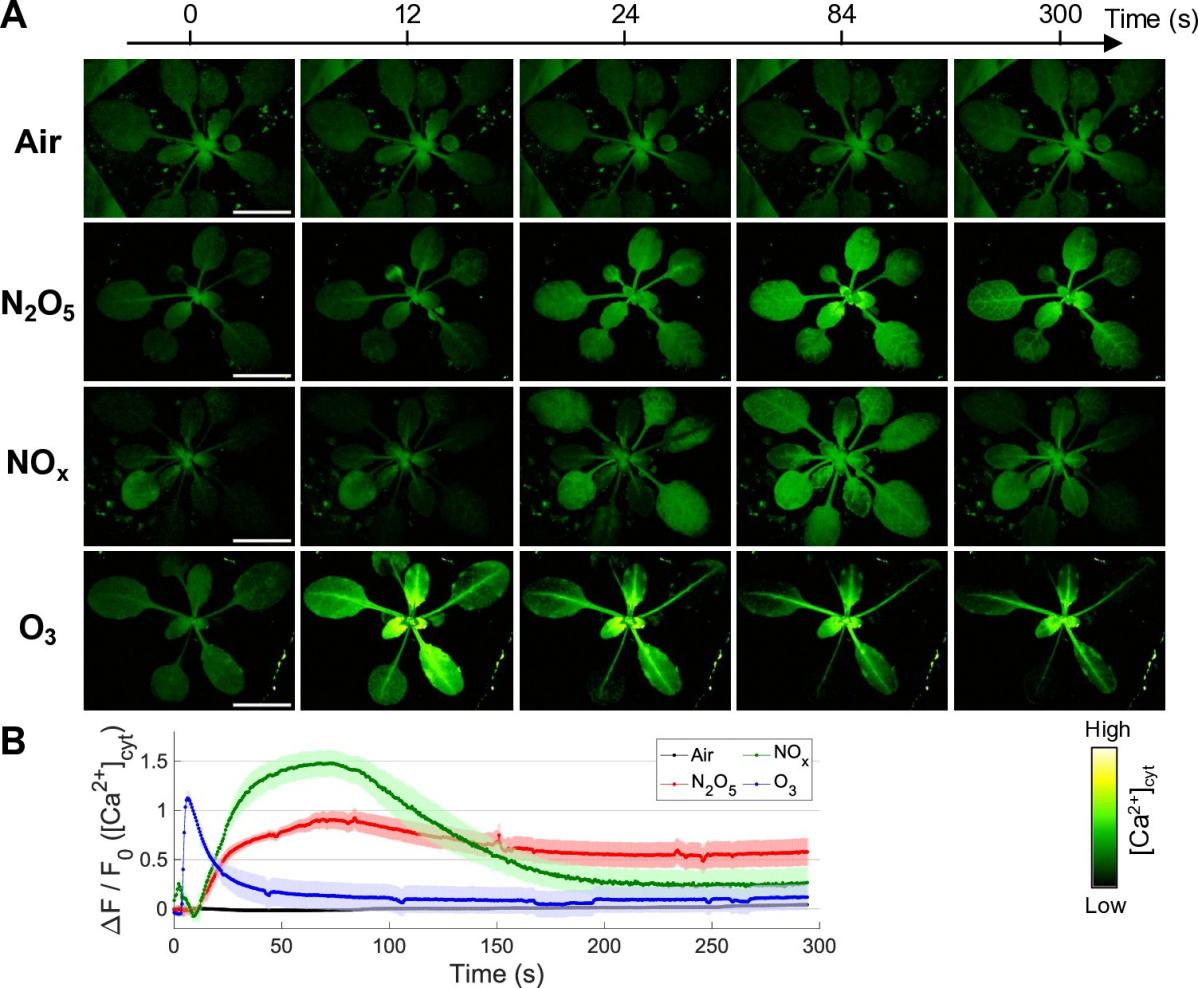
Exposure to plasma-synthesized N_2_O_5_ gas can induce a physiologically relevant [Ca^2+^]_cyt_ increase, followed by the prolonged high [Ca^2+^]_cyt_ levels in the leaf veins. (A) Time-lapse images showing changes in [Ca^2+^]_cyt_ and (B) time-course of changes in average [Ca^2+^]_cyt_ level in the three-week-old p35S-GCaMP3 (Col-0) Arabidopsis stimulated with dry air and the plasma-generated reactive gases (N_2_O_5_, NO_x_, and O_3_). Scale bars, 10 mm.

N_2_O_5_ gas exposure of the whole plant body clearly induced a gradual increase in [Ca^2+^]_cyt_ after a 10-s lag period from the start of exposure, which tended to be initiated in young leaves (Fig 3, S2 Movie), whereas no significant increase in [Ca^2+^]_cyt_ was observed in the dry-air treatment (Fig 3, S1 Movie). The average [Ca^2+^]_cyt_ in the leaves peaked at 80 s, followed by prolonged periods (at least 5 min) of lower [Ca^2+^]_cyt_ levels, although relatively high [Ca^2+^]_cyt_ levels were sustained in leaf veins. The Ca^2+^ signal dynamics after N_2_O_5_ gas exposure was found similarly after the NO_x_ gas exposure (S3 Movie), but the prolonged [Ca^2+^]_cyt_ levels in the leaf veins tended to be higher than NO_x_ gas. On the other hand, the O_3_ gas exposure resulted in a large and sharp spike in [Ca^2+^]_cyt_ during the exposure and a rapid fall concurrently with leaf curling that is the acute injury symptom (S4 Movie). Based on this result (Fig 3) in light of the absence of significant toxicity associated with the present N_2_O_5_ gas exposure (Fig 2), it was concluded that the N_2_O_5_ gas exposure induced physiologically relevant Ca^2+^ signaling.

**Fig 4:**
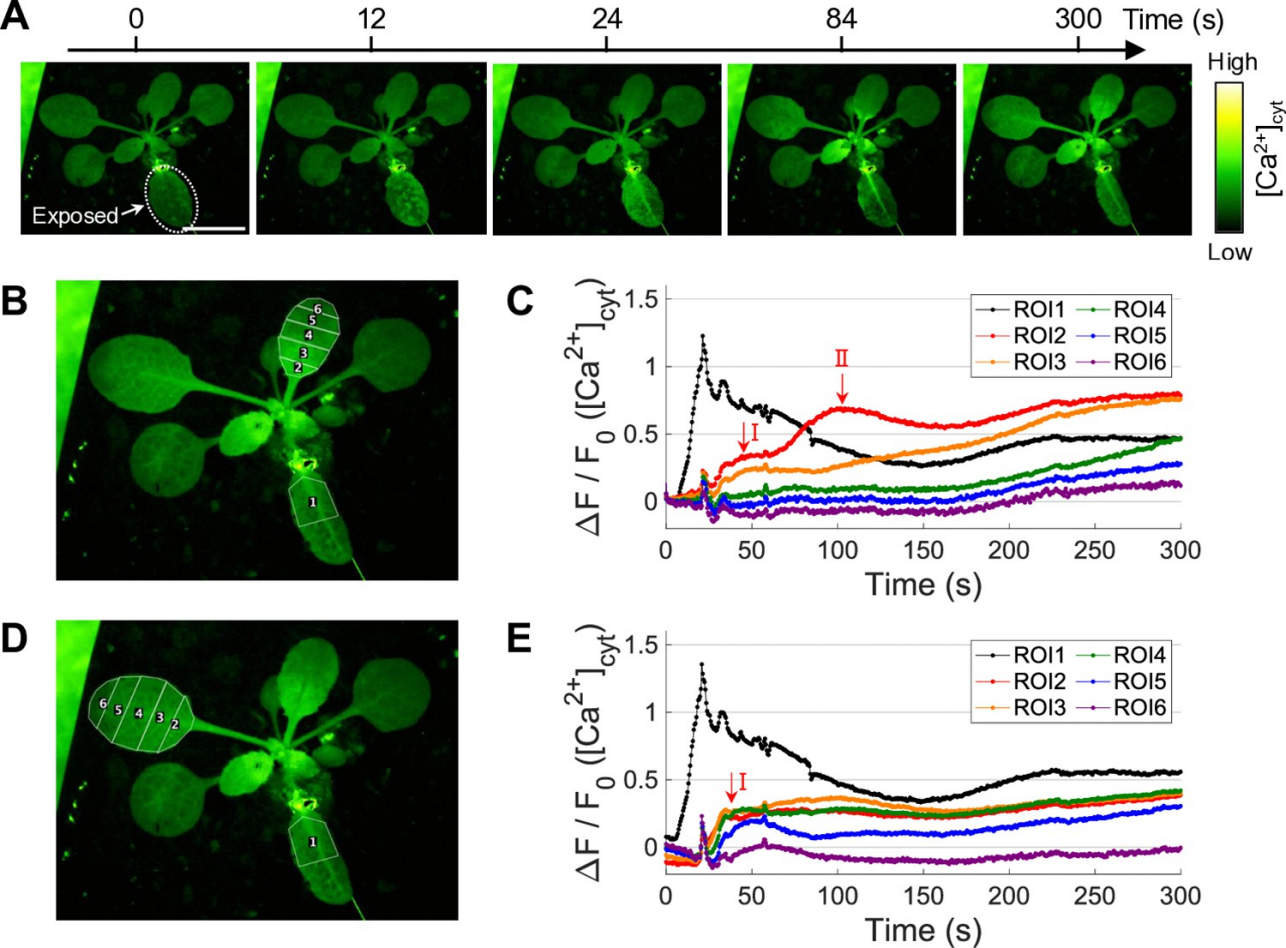
Local exposure of a leaf to plasma-synthesized N_2_O_5_ gas can trigger calcium signal propagation from the exposed leaf to the unexposed distal leaves. (A, B) Time-lapse images showing changes in [Ca^2+^]_cyt_ and (C) time-course of changes in local [Ca^2+^]_cyt_ level in a plant locally stimulated with the plasma-synthesized N_2_O_5_ gas. Scale bars, 10 mm. ROI 1 to 6 were set in (B) and mean [Ca^2+^]_cyt_ level in each ROI was plotted on (C).

To further investigate the systemic propagation of Ca^2+^ signals induced by N_2_O_5_, a targeted leaf was locally exposed to N_2_O_5_ gas (“directly exposed”), then, the non-directly exposed leaves (“indirectly exposed”) were simultaneously observed. The [Ca^2+^]_cyt_ level in the “directly exposed” leaf rose rapidly during the exposure, and the Ca^2+^ signal subsequently propagated through the petiole to “indirectly exposed” leaves (Fig 4A, S5 Movie). The rise in [Ca^2+^]_cyt_ in the directly exposed leaf was clearly larger and more rapid than that in the whole-body exposure treatment. This can be attributed to the increased dose rate of N_2_O_5_ due to the local exposure. On the other hand, the [Ca^2+^]_cyt_ increase in indirectly exposed leaves tended to be slower and more persistent compared to the whole-body exposure, although the Ca^2+^ dynamics were varied widely among the leaves. In some indirectly exposed leaves, high [Ca^2+^]_cyt_ levels were induced locally in the leaf veins, as observed in a prolonged response after the whole-body exposure treatment. Single-leaf analysis showed that, in particular, in the indirectly exposed leaves, the [Ca^2+^]_cyt_ signal propagated from the petiole to the leaf blade after a lag period of approximately 1 min (Fig 4B–4E). The Ca^2+^ wave propagations can be roughly categorized into slow and fast propagation modes in terms of propagation speed: I. ~ 200 μm/s and II. ~ 20 μm/s. In the slow propagation (Fig 4B, 4C, S5 Movie), the Ca^2+^ signal propagated from the petiole to the leaf tip while avoiding the midrib. The fast propagation speed of ~ 200 μm/s was similar to previously-reported slow Ca^2+^ waves at 100–200 μm/s observed in the mechanical wound-induced signaling [46]. Thus, these observations show that plants have a receptible mechanism to sense N_2_O_5_ and transmit this information throughout the plant body. In addition, the N_2_O_5_-induced Ca^2+^ signal dynamics were partially similar to those induced by mechanical wounding, such as high [Ca^2+^]_cyt_ level in the leaf vein and the propagation mode/velocity. This raises the hypothesis that the N_2_O_5_-induced leaf-to-leaf transmission employs a shared mechanism with the wound-triggered long-distance signal transmission leading to plant defense signaling [46].

### Defense-related gene induction induced by the APP-synthesized N_2_O_5_ gas exposure

**Fig 5:**
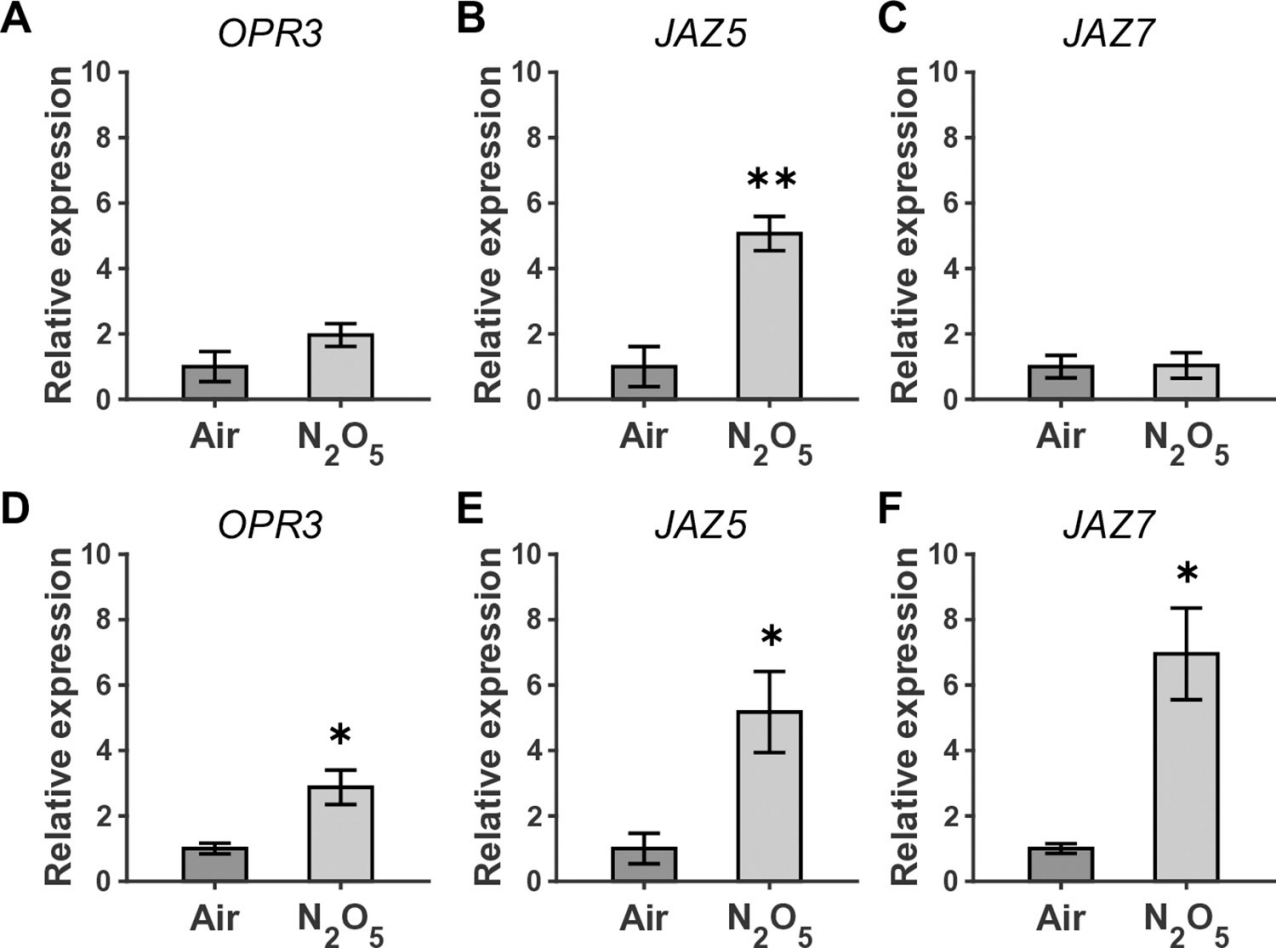
Both direct and indirect exposure to plasma-synthesized N_2_O_5_ gas can upregulate defense-related gene expression. Expression of defense-related genes, including (A, D) *OPR3*, (B, E) *JAZ5*, and (C, F) *JAZ7*, at 10 min after (A-C) direct and (D-F) indirect exposure to the plasma-synthesized N_2_O_5_ gas. (A-C) Directly-exposed leaves and (D-F) indirectly-exposed leaves were collected at 10 min and were analyzed using qRT-PCR. Statistical analysis was performed with T-test (n = 3, *p < 0.05, **p < 0.01).

To explore the possibility of a systemic defense response as plant defense signaling induced by N_2_O_5_ gas, the expression of some defense marker genes, which were reported to be transiently upregulated in the wound-triggered systemic defense response [46], was investigated in the directly and indirectly exposed leaves as shown in Fig 5. These defense-related genes are known to be associated with the plant hormone jasmonic acid (JA) pathways. The analysis time point of 10 min was determined based on the time-course changes in the gene expression in the previous study [46]. In directly exposed leaves, the expression of JA-dependent defense marker gene [jasmonate-zim-domain protein 5 (*JAZ5*)] was significantly increased at 10 min (Fig 5B), indicating that the direct exposure to N_2_O_5_ gas activates a JA signaling pathway. This is consistent with the previously reported gene ontology analysis showing the induction of many JA-responsive genes with ethylene (ET) signal activation by whole body exposure to N_2_O_5_ gas [59]. In our previous study [59], transcriptome analysis by RNA-Seq revealed that the transcripts of 828 genes increased in abundance by more than two-fold in N_2_O_5_-exposed plants, and many of them were related to JA- and ET-dependent signaling pathways. Moreover, gene analysis and inoculation tests with pathogens in Arabidopsis mutants deficient in phytohormone (*coi*1-1, *ein*2-1, and *npr*1-1) showed the importance of JA- and ET-signaling pathways in the activation of plant immunity.

Interestingly, even in indirectly exposed leaves, significant expression of oxophytodienoate-reductase 3 (*OPR3*), *JAZ5*, and jasmonate-zim-domain protein 7 (*JAZ7*) was detected (Fig 5D–5F), and this expression pattern was similar to that observed in distal leaves transmitted from a directly wounded leaf [46]. This supports the hypothesis that the N_2_O_5_ induced response employs a shared mechanism with wounding-triggered long-distance transmission, which could contribute even in the N_2_O_5_-induced signal propagation to the whole body. In addition, the expression induction of JA-responsive genes shown in Fig 5 was significantly lower in direct N_2_O_5_ exposure than in indirect exposure. This suppression may be due to higher Ca^2+^ level and some damage overall in the directly exposed leaves (Figs 3 and 4).

**Fig 6:**
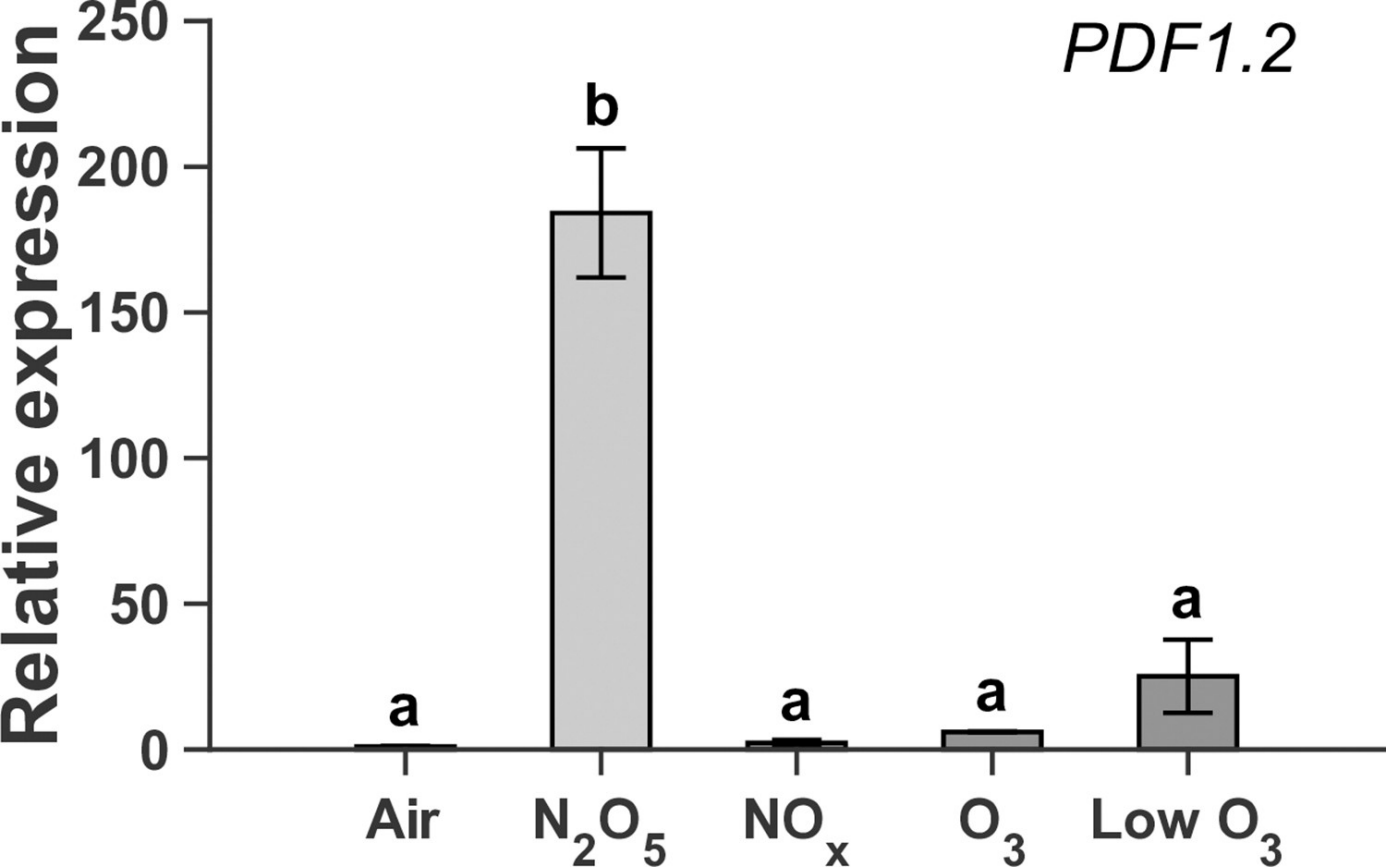
N_2_O_5_ gas specifically upregulated the plant defensin gene *PDF1*.*2*, whereas other reactive gases failed to upregulate it. *PDF1*.*2* expression in plant at 24 hours after exposure of whole body to dry air, N_2_O_5_, NO_x_, O_3_, low-density O_3_ (Low O_3_) gases. Statistical analysis was performed with Tukey-Kramer test (n = 3, p < 0.01). Treatment time was 30 s for dry air and N_2_O_5_, 15 s for dry air and NO_x_, 10 s for O_3_, corresponding to a total dose of approximately 10 μmol for N_2_O_5_, NO+NO_2_, and O_3_. Density of Low O_3_ at 7.9×10^14^ cm^-3^ was roughly adjusted to that (7.1×10^14^ cm^-3^) in the plasma-generated N_2_O_5_ gas as an impurity, and the supply for 30 s corresponded to approximately 1.3 μmol for Low O_3_.

The APP-synthesized N_2_O_5_ gas exposure also induced a very significant JA-responsive defense-related gene expression of plant defensin 1.2 (*PDF1*.*2*) gene, whereas other reactive gases such as O_3_ and NO_x_ failed to induce (Fig 6). This raises the possibility that the observed plant disease defense response is N_2_O_5_-specific, but cannot exclude the contribution of impurities in N_2_O_5_ gas. Considering the very high sensitivity to O_3_ gas in Figs 2 and 3, the contribution of O_3_ impurity in N_2_O_5_ gas to the plant disease defense response was experimentally examined with O_3_ gas density approximately ten folds lower than N_2_O_5_ gas. However, exposure to the O_3_ gas density, adjusted to that in the APP-synthesized N_2_O_5_ gas, (called “Low O_3_” in the following part of this article), did not show a significant increase in *PDF1*.*2* expression. Therefore, O_3_ alone in N_2_O_5_ gas is not responsible for inducing the observed *PDF1*.*2* expression and it is suggested that N_2_O_5_ is a key species in the induced *PDF1*.*2* expression and possibly in plant immune activation previously demonstrated through pathogen inoculation tests [59].

## Discussion

Plasma technology, which can convert air molecules into functional H_x_N_y_O_z_ with low electric power consumption, could potentially contribute to the development of a sustainable agricultural system with a low environmental impact. However, the low controllability of H_x_N_y_O_z_ synthesis using air APP and the limited understanding of plant responses to H_x_N_y_O_z_ are ones of the reasons why plasma technology has not been fully applied in agriculture. As a first step to overcome these difficulties, the present study focused on three types of N_y_O_z_ (N_2_O_5_, O_3_, and NO_x_), which can be selectively synthesized, and aimed to clarify unreported fundamental physiological responses, potentially leading to beneficial effects such as activation of plant immunity. We previously performed gene expression analysis after exposing Arabidopsis to N_2_O_5_ gas for 20 s and reported that the expression of defense response genes such as *WRKY33*, *PAD3*, and *ORA59* genes and late response *PDF1*.*2* gene was significantly induced 2 and 24 h after exposure, respectively, but these were barely or not induced in the JA signaling *coi1-1* mutant [59]. Furthermore, exposure to N_2_O_5_ gas significantly reduced the size of lesions caused by *Botrytis cinerea* infection in wild type, *ein2-1*, and *npr1-1* plants compared to air controls, whereas no significant difference was observed in *coi1-1* [59]. Combining these previous studies with the results of the present study, we deduce that APP-synthesized N_2_O_5_ gas induces systemic Ca^2+^ signaling throughout the plant body, which can lead to the activation of JA signaling and plant disease responses, even when gas treatment is concentrated in a single leaf.

The APP-synthesized N_2_O_5_ gas was less toxic to plants than O_3_ gas (Fig 2), but more toxic than NO_x_ gas. Exposure treatments with NO_x_ gas did not cause significant damage to plants, even in an overdose range (> 100 μmol) where N_2_O_5_ gas caused the observed damage [59, 62]. This N_2_O_5_ gas-induced damage might be attributed to the low density of O_3_ as an impurity, but the toxicity symptoms (*e*.*g*., leaf yellowing rather than less leaf curling) seemed to differ from those of O_3_ exposure. Thus, higher doses of N_2_O_5_ may negatively affect plant health in a different manner from O_3_. In plants exposed to O_3_, a hypersensitivity response accompanied by program cell death is activated [49, 63]. The N_2_O_5_ gas exposure at the moderate dose induced a transient [Ca^2+^]_cyt_ rise, the pattern of which was similar to that in NO_x_ gas (Fig 3), but NO_x_ gas did not induce *PDF1*.*2* gene (Fig 6). Furthermore, the local N_2_O_5_ gas exposure of a single leaf triggered the systemic signal transmission (Fig 4), resulting in the activation of JA signaling in the distal leaves (Fig 5). High expression levels of *PDF1*.*2* at 24 hours were found only in the N_2_O_5_ gas-treated samples among the given reactive gas exposure including the Low O_3_ (Fig 6), supporting that N_2_O_5_ is a key species.

The N_2_O_5_ gas-induced systemic Ca^2+^ signaling and the expression pattern of the defense-related genes in indirectly exposed leaves were partially similar to those in the physical wound-induced response, suggesting a shared transmission mechanism. Unlike the wound-induced response, the initial point of N_2_O_5_ attack remains still unknown, and elucidation of this is an important future challenge. The [Ca^2+^]_cyt_ increase observed in this study is a relatively early response within 10 seconds of the exposure start, and is therefore an important clue toward the identification of the initial action site. A close-up observation of an N_2_O_5_ gas-exposed leaf showed that Ca^2+^ signaling was initiated in scatteredly distributed small spots (S2 Fig). Considering the reactivity acquisition of N_2_O_5_ on the wet surface through intermediate species (e.g., [NO_2_^+^·NO_3_^−^]_aq_, NO_2_^+^_aq_) generation by reaction with water, the first contact of N_2_O_5_ with moist tissues, which are exposed to open air or accessible through pores such as stomata, would be key.

Two of the most studied types of induced resistance are systemic acquired resistance (SAR) and induced systemic resistance (ISR) [64]. In the SAR pathway, salicylic acid (SA) plays as a key signaling molecule and genes encoding pathogenesis-related (PR) proteins are often regarded as marker genes. It is well known that exposure of plants to O_3_ at relatively low-density ranges (< ppm) and over the long term (> hours) can induce the accumulation of SA and pathogenesis-related 1 (*PR1*) gene expression, which are important for the SAR activation pathway [47, 49]. In the present study, the induction of *PR1* expression by N_2_O_5_ gas exposure for 10 s and 30 s was not significant while *PDF1*.*2* expression was prominently increased (S3 Fig). On the other hand, ISR reportedly requires JA and ET hormonal signaling pathways. The significant induction of JA-related gene expression in the indirectly exposed leaves (Fig 5), together with the fact of JA/ET signaling pathway activation shown in the previous study [59], suggests that N_2_O_5_ gas exposure induces an ISR-like systemic response. Some reports discuss SA/JA crosstalk, where SA- and JA-mediated signaling interact with each other antagonistically or synergistically depending on the conditions [64–66]. Therefore, a clear dichotomy between SAR and ISR might be difficult, but supposing that N_2_O_5_ primarily induces JA-related signaling and SA/JA are antagonistically interfering, no significant expression of *PR1* by N_2_O_5_ gas containing O_3_ as an impurity could be consistently explained.

The concept of controlling and supplying air-derived H_x_N_y_O_z_ including N_2_O_5_ for plant disease management and post-harvest processes is very promising as one of plasma agricultural applications, but the progress requires further elucidation of action mechanisms of individual or mixed H_x_N_y_O_z_ on plants. A better understanding at the molecular level of the sensing and response mechanisms of plants to unknown H_x_N_y_O_z_ would lead to the emergence of new applications. We anticipate that results in the present study will inspire many researchers in not only the plasma applied physics and chemistry but also plant physiology and other biological fields, leading to the progress in the field of plasma agriculture.

## Methods

### Air plasma system for on-site generation of reactive species (N_2_O_5_, NO/NO_2_, O_3_)

The reactive gases were synthesized from air using a device developed in a previous study [58]. Briefly, the selective generation of gaseous N_2_O_5_ in extremely dry air (typically below 10 ppm of H_2_O) was achieved by mixing reactive gases from two independent plasma reactors: 1. low gas temperature (LT) reactor for selective O_3_ generation and 2. high gas temperature (HT) reactor for selective NO/NO_2_ generation. The setups for selective generation of N_2_O_5_, NO/NO_2_, and O_3_ were called “N_2_O_5_ mode,” “NO_x_ mode,” and “O_3_ mode,” respectively. The mode can be easily changed by electric switching for the HT and LT plasma reactors. The total gas flow rate was set at 2 L/min. Further details of the device/method can be found in our previous study [58].

The composition of reactive species was analyzed using a Fourier Transform Infrared (FTIR) spectrometer (FT/IR-6100TUK; JASCO, Japan) equipped with a gas cell with five-meter-long optical path at 45°C. Reactive species’ densities were quantified using the least square error fitting of the measured spectra with synthetic spectra, composed from absorption cross sections obtained from the HITRAN database [67].

### Plant cultivation and exposure of plants to reactive gases

The seeds of *A*. *thaliana* (Col-0 accession) were obtained from the Nottingham Arabidopsis Resource Centre (https://arabidopsis.info/). The transgenic line of *A*. *thaliana* expressing GCaMP3 has been described previously [46]. In brief, the GCaMP3 fragment, gifted by Loren Looger (Addgene plasmid #22692), was cloned into a pAN19 vector containing the CaMV35S promoter (p35S). The entire cassette of p35S::GCaMP3 NOSt was inserted into the plant binary vector, pBIN20. This construct was transformed into *A*. *thaliana*. A voucher specimen of this material has not yet been deposited in a publicly available herbarium. *A*. *thaliana* plants were cultured in chambers at 23°C with an 16 h-light/8 h-dark photoperiod under fluorescent lamps (100 μmol m^−2^ s^−1^). All plant experiments described in this study were performed with relevant institutional, national and international guidelines and legislation.

Reactive gases were generated by an air plasma system in each mode and supplied to 2- to 3-week-old Arabidopsis plants through a polytetrafluoroethylene (PTFE) tube with an internal diameter of 4 mm. Two-week-old plants were used for experiments to measure fresh weight after 2 weeks of gas exposure, and 3-week-old plants were used for analyses of cytoplasmic Ca^2+^ signals and transient gene expression. For whole-body exposure, each *A*. *thaliana* plant pot was placed 10 mm downstream from the tube exit. For local exposure, a single leaf was selectively exposed to the N_2_O_5_ gas by covering *A*. *thaliana* plant with a transparent film with a small hole.

### Real-time [Ca^2+^]_cyt_ imaging system

*A*. *thaliana* plants expressing genetically-encoded Ca^2+^ indicators were imaged with a fluorescence stereo microscope (SMZ18; Nikon, Japan) equipped with a CMOS camera (DP74; Olympus, Japan). The GFP-based Ca^2+^ indicator, GCaMP3, was excited using a mercury lamp (Intensilight C-HGFI; Nikon, Japan) with a 470/40 nm excitation filter and the green fluorescent signal passing through a 535/50 nm filter was acquired every 0.52 s. For local exposure, a transparent film with a small hole was used and [Ca^2+^]_cyt_ observation of whole body with targeted treatment of a single leaf was realized.

The GCaMP3 signals were analysed over time at several regions of interest (ROIs) using ImageJ. Changes in [Ca^2+^]_cyt_ were expressed as ΔF/F = (F − F_0_)/F_0_, where F_0_ represents the average baseline fluorescence determined by the average of F over the 6 frames before the exposure treatment [60].

### Gene expression analysis by qRT-PCR

The analysis time points of 10 min for *OPR3*, *JAZ5*, and *JAZ7* genes [46] and 24 hours for *PDF1*.*2* and *PR1* gene [59] were determined based on the time-course changes in gene expression in the previous studies. Total RNA was extracted from leaves for whole-body exposure or directly-exposed/indirectly-exposed leaves for local exposure with Trizol Reagent (Invitrogen). Finally, 500 ng of total RNA (A260/280 ratio 1.8 <, measured with NonoVue (GE Healthcare) from each sample was used for cDNA synthesis with a PrimeScript II 1st strand cDNA Synthesis Kit and random primers (TaKaRa Bio). Real-time quantitative RT-PCR was performed with SYBR Premix Ex Taq II (TaKaRa Bio), in a CFX96 Real-Time System (Bio-Rad Laboratories), with the forward and reverse primers in Table 1. For each sample condition, three additional technical iterations were performed for each of the three independent biological samples, and their means and standard deviations were calculated. The real-time RT-PCR data was normalized to *Tubulin 2/3* as an internal control [68].

**Table 1:** Primers used in this study.

Gene name	Forward (5’-3’)	Reverse (5’-3’)
*OPR3*	CGTTTTACACTCAAGATCCAGTTG	ATTATCAAACTCAGAGGCGGG
*JAZ5*	TCATCGTTATCCTCCCAAGC	CACCGTCTGATTTGATATGGG
*JAZ7*	GATCCTCCAACAATCCCAAA	TGGTAAGGGGAAGTTGCTTG
*PDF1*.*2*	ATGCATGCAAGAATCAGTGC	AATACACACGATTTAGCACC
*PR1*	AGCCTATGCTCGGAGCTACG	ACCCCAGGCTAAGTTTTCCC
*Tubulin 2/3*	CCAGCTTTGGTGATTTGAAC	CAAGCTTTCGGAGGTCAGAG

### Quantification and statistical analysis

All analyses were performed in at least biological triplicate for each sample. Statistical analyses were performed using Student’s t-test or Tukey-Kramer test. p values less than 0.05 were classed as statistically significant.

## Supporting information

S1 Table Reactive species’ composition of reactive gases at N_2_O_5_ mode, NO_x_ mode, O_3_ mode, and low O_3_ mode. (DOCX)

S1 Fig Typical images of plants at 2 weeks after exposure to dry air, N_2_O_5_, and O_3_ gases. Treatment time is 30 s for dry air and N_2_O_5_, 10 s for O_3_, corresponding to total dose of approximately 10 μmol for N_2_O_5_ and O_3_.
(PDF)

S2 Fig The close-up observation and analysis of an N_2_O_5_ gas-exposed leaf. (A) Close-up time-lapse images showing changes in [Ca^2+^]_cyt_ in the Arabidopsis plant stimulated with the N_2_O_5_ gas. Scale bars, 1 mm. (B) The difference images from the fluorescence image at 09 s. (C) Box plot of size distribution of bright spot extracted from the fluorescence image at 12 s.
(PDF)

S3 Fig (A) *PDF1*.*2* and (B) *PR1* expression in plant at 24 hours after exposure of whole body to dry air and N_2_O_5_ gases. Treatment time is 30 s for dry air and, 10 s and 30 s for N_2_O_5_.
(PDF)

S1 Movie Typical time-lapse movie showing changes in [Ca^2+^]_cyt_ after the whole-body exposure to dry air. (AVI)

S2 Movie Typical time-lapse movie showing changes in [Ca^2+^]_cyt_ after the whole-body exposure to N_2_O_5_ gas. (AVI)

S3 Movie Typical time-lapse movie showing changes in [Ca^2+^]_cyt_ after the whole-body exposure to NO_x_ gas. (AVI)

S4 Movie Typical time-lapse movie showing changes in [Ca^2+^]_cyt_ after the whole-body exposure to O_3_ gas. (AVI)

S5 Movie Typical time-lapse movie showing changes in [Ca^2+^]_cyt_ after the local exposure to N_2_O_5_ gas. (AVI)
