# Supplementary material for: Induction of systemic resistance through calcium signaling in Arabidopsis exposed to air plasma-generated dinitrogen pentoxide
Source: PLoS One. 2025 Feb 6;20(2):e0318757. doi: 10.1371/journal.pone.0318757 (PMC11801567; doi:10.1371/journal.pone.0318757)
Supplement: S1 Table — (DOCX) [file pone.0318757.s001.docx]

| **Species** | **N_2_O_5_ mode** | **NO_x_ mode** | **O_3_ mode** | **Low O_3_ mode** |
| --- | --- | --- | --- | --- |
| N_2_O_5_ | 5.6 × 10^15^ cm^-3^  (~230 ppm) | < LOD | 2.1 × 10^14^ cm^-3^  (~8.4 ppm) | < LOD |
| NO | < LOD | 8.6 × 10^15^ cm^-3^  (~350 ppm) | < LOD | < LOD |
| NO_2_ | 7.6 × 10^14^ cm^-3^  (~31 ppm) | 2.4 × 10^15^ cm^-3^  (~99 ppm) | < LOD | < LOD |
| O_3_ | 7.1 × 10^14^ cm^-3^  (~29 ppm) | < LOD | 1.9 × 10^16^ cm^-3^  (~770 ppm) | 7.9 × 10^14^ cm^-3^  (~32 ppm) |
