# Supplementary figures and images for: Induction of systemic resistance through calcium signaling in Arabidopsis exposed to air plasma-generated dinitrogen pentoxide
Source: PLoS One. 2025 Feb 6;20(2):e0318757. doi: 10.1371/journal.pone.0318757 (PMC11801567; doi:10.1371/journal.pone.0318757)

2 weeks after

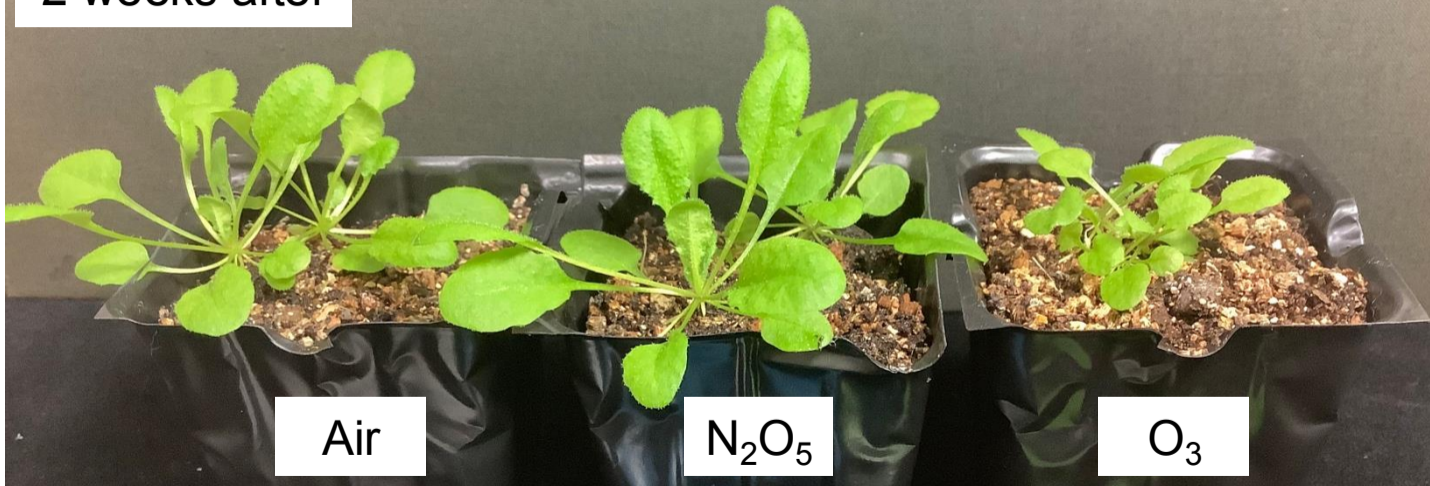

Air

$\text{N}_2\text{O}_5$

$\text{O}_3$

Supplement: S1 Fig — Treatment time is 30 s for dry air and N2O5, 10 s for O3, corresponding to total dose of approximately 10 μmol for N2O5 and O3. (PDF) [file pone.0318757.s002.pdf]

**A**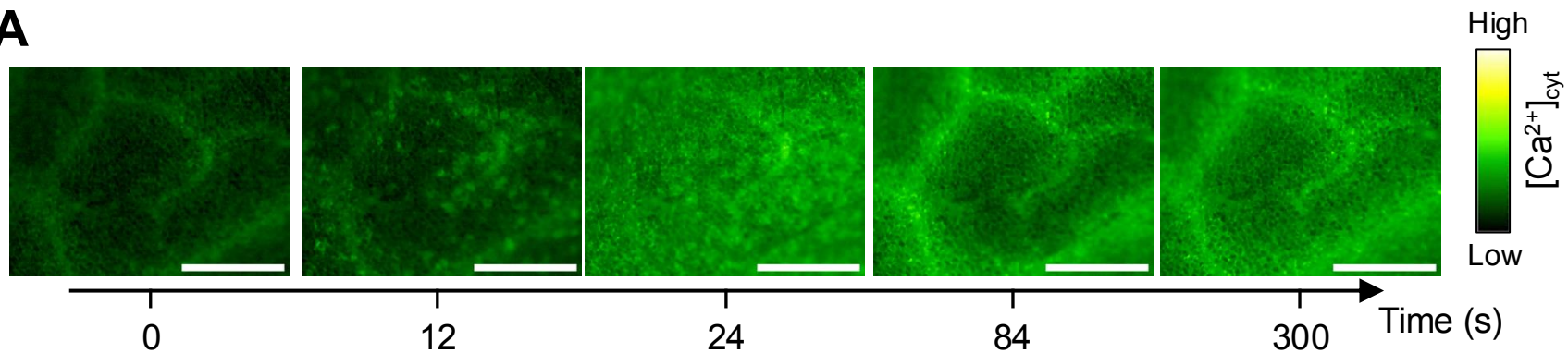**B**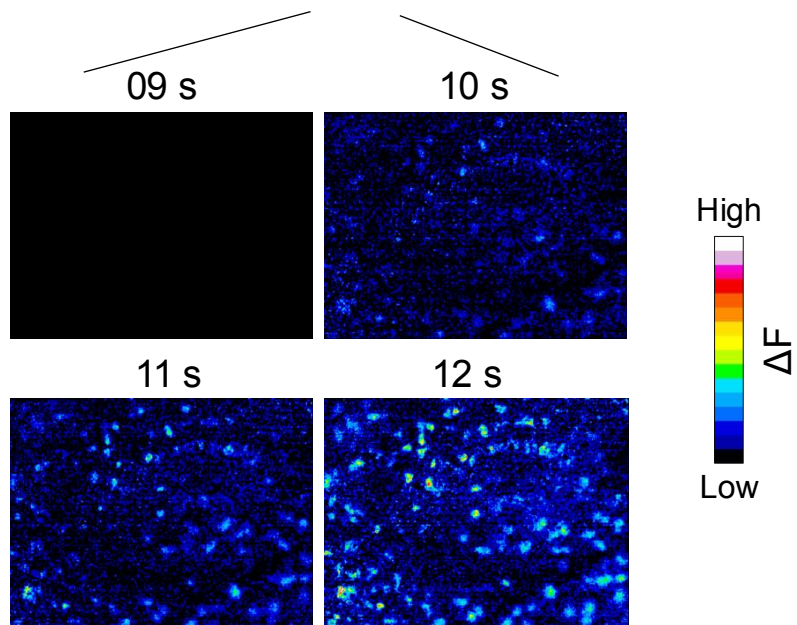**C**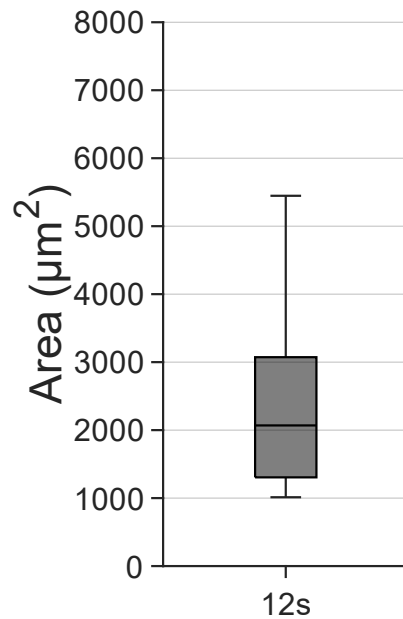

Supplement: S2 Fig — (A) Close-up time-lapse images showing changes in [Ca2+]cyt in the Arabidopsis plant stimulated with the N2O5 gas. Scale bars, 1 mm. (B) The difference images from the fluorescence image at 09 s. (C) Box plot of size distribution of bright spot extracted from the fluorescence image at 12 s. (PDF) [file pone.0318757.s003.pdf]

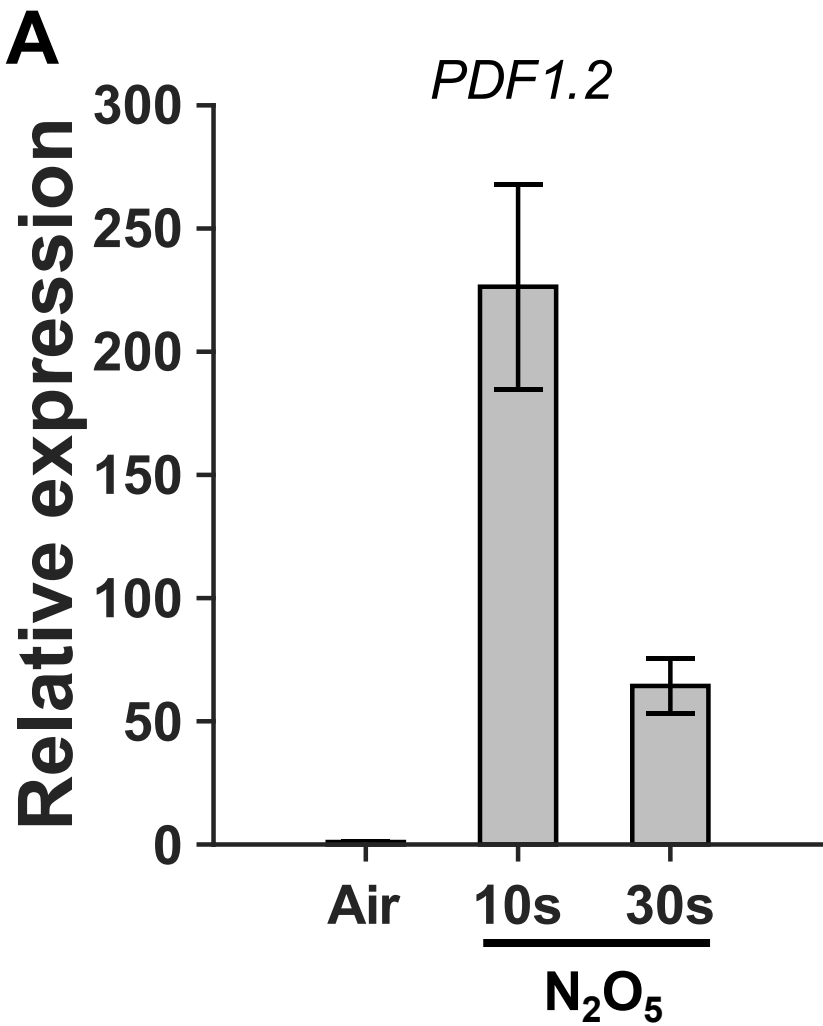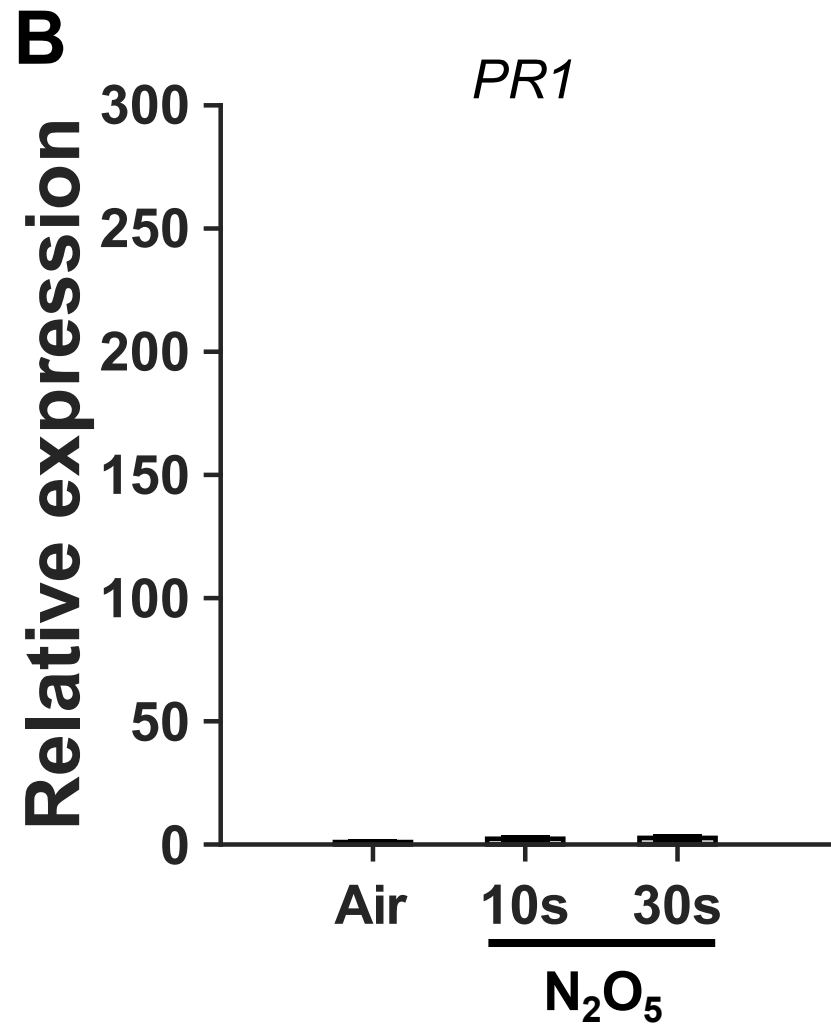

Supplement: S3 Fig — (A) PDF1.2 and (B) PR1 expression in plant at 24 hours after exposure of whole body to dry air and N2O5 gases. Treatment time is 30 s for dry air and, 10 s and 30 s for N2O5. (PDF) [file pone.0318757.s004.pdf]
